# Cardiac dysfunction in severe pediatric acute respiratory distress syndrome: the right ventricle in search of the right therapy

**DOI:** 10.3389/fmed.2023.1216538

**Published:** 2023-08-16

**Authors:** Lece Webb, Luke Burton, Ananya Manchikalapati, Priya Prabhakaran, Jeremy M. Loberger, Robert P. Richter

**Affiliations:** ^1^Division of Pediatric Critical Care Medicine, Department of Pediatrics, University of Alabama at Birmingham, Birmingham, AL, United States; ^2^Division of Pediatric Critical Care, Department of Pediatrics, University of Texas Southwestern Medical Center, Dallas, TX, United States

**Keywords:** children, pediatric acute respiratory distress syndrome (PARDS), right ventricular (RV) dysfunction, echocardiography (Echo), extracorporeal membrane oxygenation (ECMO)

## Abstract

Severe acute respiratory distress syndrome in children, or PARDS, carries a high risk of morbidity and mortality that is not fully explained by PARDS severity alone. Right ventricular (RV) dysfunction can be an insidious and often under-recognized complication of severe PARDS that may contribute to its untoward outcomes. Indeed, recent evidence suggest significantly worse outcomes in children who develop RV failure in their course of PARDS. However, in this narrative review, we highlight the dearth of evidence regarding the incidence of and risk factors for PARDS-associated RV dysfunction. While we wish to draw attention to the absence of available evidence that would inform recommendations around surveillance and treatment of RV dysfunction during severe PARDS, we leverage available evidence to glean insights into potentially helpful surveillance strategies and therapeutic approaches.

## Introduction

Acute respiratory distress syndrome (ARDS) in children, or pediatric ARDS (PARDS), is a common but severe manifestation of a host of insults to the respiratory system of a child that carries a significant risk of morbidity and mortality (1). As in a patient of any age, ARDS involves direct and/or indirect mechanisms that disrupt the protective surface tension along the apical surface of alveolar cells, flood alveoli with cellular debris, and promote pulmonary interstitial disruption through leukocyte recruitment and local microvascular endothelial leakage. Together, the resultant lung pathobiology leads to a clinical syndrome of respiratory system failure with hypoxemia and hypercapnia that can cascade into multiorgan failure and late death (2).

In our experience, right ventricular (RV) dysfunction and eventual failure is an important, and often occult, driver of multiorgan failure in the setting of PARDS. Though RV dysfunction is a well-known phenomenon described in adult ARDS literature, evidenced by various reviews on the topic in recent years (3–6), there is a dearth of literature on the topic in children. The purpose of this narrative review is thus two-fold. First, we wish to bring a greater awareness of this under-recognized disease process to the pediatric critical care community. Second, we are issuing a clarion call for pediatric researchers to improve our understanding of the incidence of and mechanisms driving this disease. It is our hope that by calling greater attention to the often-neglected right heart, we may substantially improve outcomes in children with PARDS.

## PARDS definition and evidence-based management

Though operational definitions for ARDS have existed for adults for decades (7–9), their validity in children had not been formally tested and thus remained limited in this population. Researchers within the Pediatric Acute Lung Injury and Sepsis Investigators network convened the Pediatric Acute Lung Injury Consensus Conference (PALICC) to gain consensus on the first pediatric-focused definition of ARDS (10). Together, the collaborators acknowledged the essential role that mean airway pressure plays in driving oxygenation and thus implemented the oxygenation index to stratify PARDS instead of a PaO_2_/F_I_O_2_ ratio. The Consensus Conference also recognized that use of arterial oxygen sampling is not homogeneous across pediatric intensive care units (PICU) and thus incorporated oxyhemoglobin data from pulse oximetry into the determination of PARDS severity when PaO_2_ data are unavailable. The PALICC experts simplified the radiological criteria for PARDS to any radiographic evidence of alveolar disease rather than “bilateral opacifications” on chest imaging as recommended in the Berlin criteria. Unique to the PALICC definition, PARDS could be described in specific pediatric populations with pre-existing comorbidities such as chronic lung disease and cyanotic heart disease.

Lung protective ventilation using low tidal volume and higher positive end-expiratory pressure-to-fraction of inspired oxygen (PEEP/F_I_O_2_) ratios became standard management of ARDS in adults following the first ARDS Network trial in 2000 (11). Until the last decade, PARDS management subsisted without consensus recommendations and remained at the discretion of individual PICU providers. In 2015 and largely informed by the ventilator strategy described by the ARDS Network, PALICC published the first consensus recommendations for the management of PARDS (10). This has been followed by a very recent update published in February 2023 focusing on emerging evidence and resource-limited settings but generally carried forward the same recommendations as described in 2015 (12).

**Figure 1:**
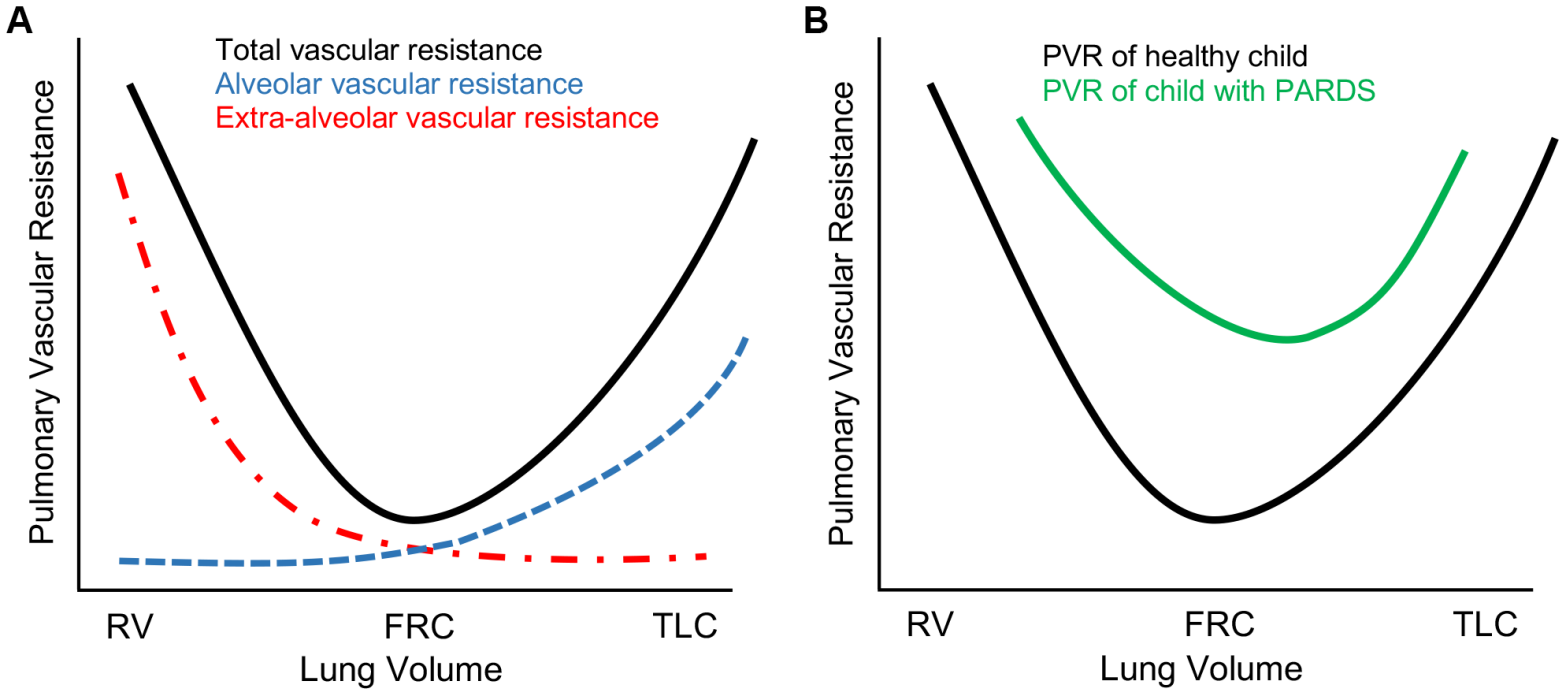
Pulmonary vascular resistance (PVR) changes in severe pediatric acute respiratory distress syndrome (PARDS). **(A)** Schematic representation of total PVR as a function of lung volume in a healthy pediatric lung. PVR is lowest at functional residual capacity (FRC) and highest either at residual capacity (RV) where PVR is elevated due to resistive changes in extra-alveolar vasculature or total lung capacity (TLC) where PVR is elevated due to resistive changes in alveolar vasculature. **(B)** In severe PARDS, PVR is globally elevated secondary to a complex combination of the following: (1) lung architectural heterogeneity from regional atelectasis, alveolar overdistention, or parenchymal cystic changes; (2) hypoxia-, hypercapnia-, and acidemia-mediated vasoconstriction; (3) parenchymal inflammatory changes with associated pulmonary edema.

For the typical patient with severe PARDS, standard ventilator management consists of low tidal volume ventilation (4–6 mL/kg of ideal body weight) and higher PEEP/F_I_O_2_ ratios with the express intent of limiting plateau pressure to below 28 cmH_2_O, limiting driving pressure (defined as plateau pressure minus PEEP) to less than 15 cmH_2_O, and preserving functional residual capacity (FRC) by preventing atelectasis (13). However, PARDS manifests along a spectrum of phenotypes and severity (14). Moreover, the respiratory system of a critically ill child with PARDS must managed within the context of the entire patient, most especially considering the interactions between intrathoracic pressure changes and cardiovascular function (see next section for more detail). For example, to achieve the afore-mentioned ventilatory targets, it is common practice to accept permissive hypoxemia and hypercapnia for a patient with severe PARDS in an effort to limit ventilator-induced lung injury with recommended lower limits of oxygen saturations and arterial pH of 88% and 7.20, respectively (12, 13). However, the ensuing hypoxemia, hypercapnia, and acidemia, in tandem with the disturbance in normal lung architecture from regional atelectasis or alveolar overdistention, increases pulmonary vascular resistance (PVR) and RV afterload (Figure 1) that may prove harmful for children with limited capacity to handle acute changes in RV end-diastolic pressure (RVEDP) or volume (RVEDV). Though the authors of the PALICC-2 guidelines acknowledge the potential for lung-protective interventions to impact biventricular function, further research is needed to more clearly define ideal management of the right heart concurrent with lung-protective strategies (12, 15).

## Right heart dysfunction during PARDS

### Anatomy and physiology of the pediatric RV in health and disease

The human heart undergoes developmental changes throughout childhood that are important to consider in the management of PARDS (see Table 1). *In utero*, high PVR facilitates the redirection of systemic and placental venous return away from the lung through either the ductus arteriosus or the foramen ovale for eventual ejection to the systemic circulation. As the morphologic RV (assuming situs solitus with levocardia and D-looped ventricles) is conditioned by elevated PVR *in utero*, RV and left ventricle (LV) wall thicknesses are nearly identical at birth (16). As the PVR drops postnatally with a neonate’s first breaths, RV afterload rapidly declines. This permits the gradual reconditioning of the RV in a low pressure environment that results in thinning of the RV wall mass over the ensuing weeks-months of infancy.

**Table 1:** Differences in myocardial cellular anatomy and cardiopulmonary physiology between neonates and older children/adults.

Variable	Neonate/young infant	Older child/adult	Effect on right ventricle
Pulmonary vascular resistance	↑	↓	Afterload, myocardial stress (systolic function)
Cardiac myocyte connective tissue-to-contractile protein ratio	↑	↓	Contractility (systolic function) Wall stiffness (diastolic function)
Myofibril organization in cardiac myocyte	Disorganized, not uniformly linear	Mature, linear alignment	Contractility (systolic function)
Development of transverse tubules and sarcoplasmic reticula	Underdeveloped	Mature	Neonate: reliance on extracellular sources of Ca^2+^ for myofibril contraction Older child: well-synchronized Ca^2+^-induced Ca^2+^ release from sarcoplasmic reticula in response to cardiomyocyte membrane depolarization, facilitating coordinated myofibril contraction
Myocardial β_1_-receptor expression	↓	↑	Contractility (systolic function)

At a cellular level, myocardial fibers of the neonatal and young infant’s heart globally have higher connective tissue-to-contractile protein ratios with generally fewer and less organized myofibrils present per cardiomyocyte (17). This myofibril anatomy compromises the ability of the cardiomyocyte to both contract and to relax, leading to a state of minimal systolic and diastolic reserve. The transverse tubules and sarcoplasmic reticula overlying myofibrils within cardiomyocytes are also immature in the young infant, further limiting the calcium-dependent inotropic capacity of the infant’s myocardium and rendering the myocardium reliant upon extracellular calcium sources for sarcomeric contraction. Moreover, the neonatal heart has a higher preponderance of parasympathetic innervation with lower β-adrenergic receptor expression compared to older children or adults, limiting RV and LV contractile reserve.

The density and anatomic arrangement of RV fibers also contribute to the limited contractile reserve of the RV. In contrast to the LV that is comprised of three myocardial fiber layers with complex alignment permitting torsional constriction of the LV cavity, the RV is generally made up of only superficial and deep layers of muscle fibers (18). The superficial myofibers are predominantly transversely oriented, blending into the superficial myocardial layer of the ventricular septum and LV, while the deeper myofibers are more longitudinally aligned (18). Given the lower postnatal wall stress perceived by the RV, each myocardial fiber within the RV carries a substantially reduced number of mitochondria (19). These factors together limit RV contractile capacity and metabolic reserve at baseline in both neonates and younger children.

In older children as in adults, the RV generally has a greater capability of tolerating sudden myocardial demands (e.g., increases in preload and/or afterload) than neonates through elevations in heart rate, contractility, and stretch to accommodate the increased RVEDV. Moreover, extrapolating from canine models of acute afterload changes to the ventricles, LV contractility may contribute 20%–40% of RV output in older children through ventricular tethering (20). In neonates, such a demand on the RV is not as well tolerated. In addition to the cellular and anatomic differences in the neonatal myocardium described above, the resting heart rate of neonates is typically higher than older children. Thus, the “therapeutic window” by which neonatal heart rates can elevate to generate a compensatory increase in cardiac output before tachycardia limits ventricular filling is narrower than older children. Additionally, the PVR in some infants may remain elevated for the first several months of life, increasing basal afterload on the RV. In children with persistently elevated PVR from birth as seen in bronchopulmonary dysplasia or congenital heart diseases with pulmonary over-circulation, the poorly compliant RV is able to gradually adapt to the higher levels of wall stress through myocardial hypertrophy. However, this compensatory mechanism comes at the cost of further limiting RV diastolic function (21).

Given the limited contractile reserve of the RV in an older child, acute increases in RVEDV are initially tolerated through modest dilation of the relatively compliant RV wall. As greater stress is placed on the RV myocardium through increasing afterload and/or preload and myofibrils are stretched further, myosin-actin interactions are reduced and systolic function becomes embarrassed (extreme of the Frank–Starling relationship). The reduction in RV contractility is thus unable to respond to the increased preload by raising, or even maintaining, stroke volume. The ensuing acute RV dilation can cause a precipitous compression of the LV through ventricular interdependence. In combination with diminished pulmonary venous return due to limited RV output and reduced pulmonary blood flow, LV compression can result in an embarrassment to systemic cardiac output—a process termed acute cor pulmonale (ACP). The combination of increased RV wall stress and decreased systemic cardiac output can result in coronary ischemia that further reduces RV systolic function that can culminate in cardiac arrest.

**Figure 2:**
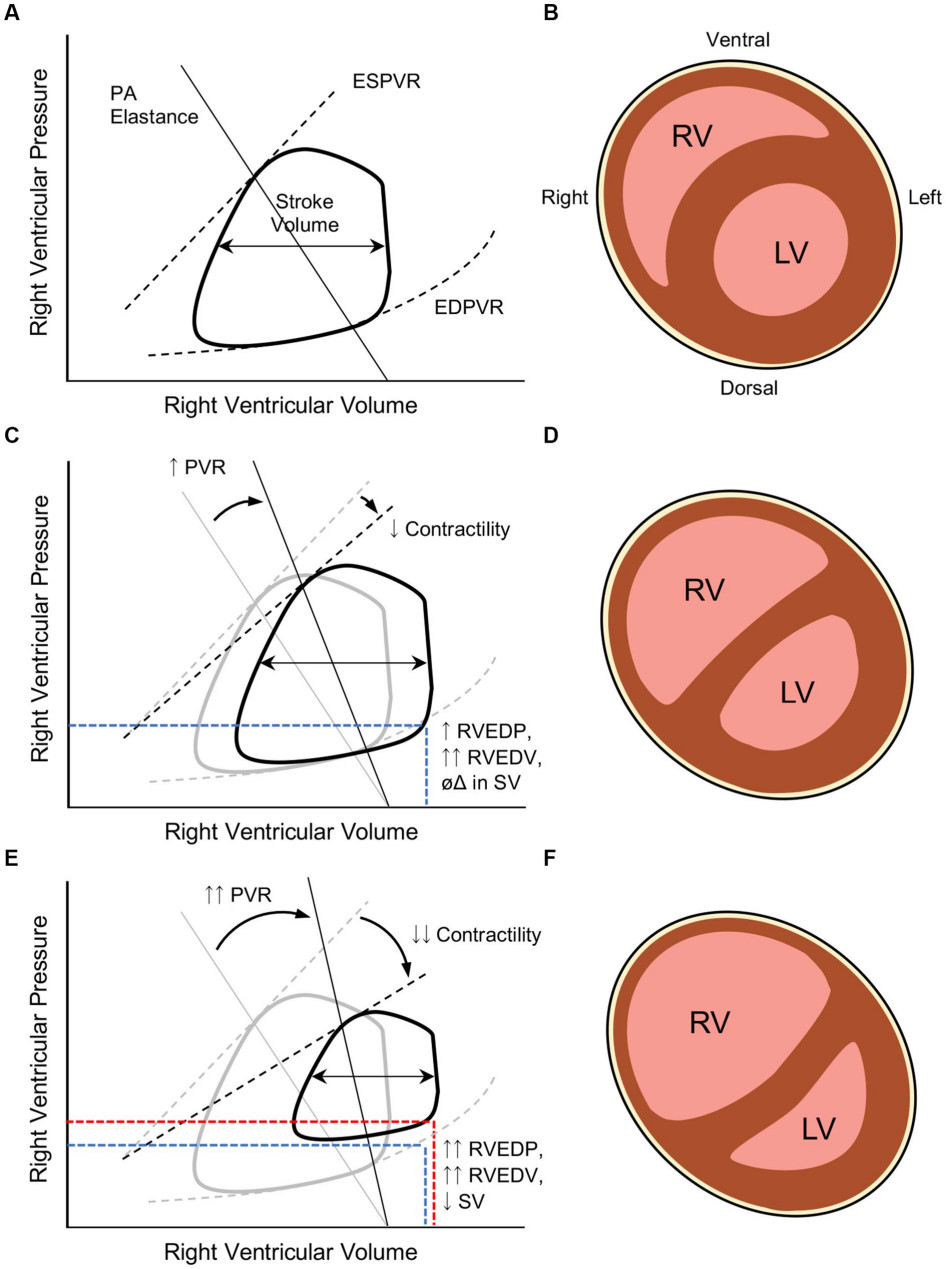
Pathophysiologic changes in right ventricular (RV) systolic and diastolic function during severe pediatric acute respiratory distress syndrome (PARDS). **(A)** Normal RV end-systolic and end-diastolic pressure-volume relationships (ESPVR and EDPVR, respectively) in a healthy child as a function of pulmonary arteriolar (PA) elastance that generates a given stroke volume (difference in RV end-systolic and end-diastolic volumes, or RVESV and RVEDV). Adapted from Brener et al. (22). **(B)** After the first several months of life, the RVEDP is significantly lower than the LVEDP in a healthy child, leading to the RV taking a more crescentic shape around the LV (cartoon transverse cross-section of a situs solitus heart with D-looped ventricles). **(C)** In severe PARDS, increases in PVR result in higher RV afterload that, in a developing heart, can precipitate RV systolic dysfunction that manifests with impaired contractility. The result of these pathologic changes result in higher RVEDP and RVEDV; however, stroke volume (SV) is preserved. **(D)** Elevated RVEDP and RVEDV begins to dilate the RV and compete against LVEDP, leading to ventricular septal flattening. **(E)** In the setting of RV failure, the RV does not adapt to the higher RVEDV and begins to manifest lower stroke volumes. **(F)** The much higher RVEDP begins to reach or surpass LVEDP and impose upon LVEDV. The diminished LV preload from both a reduction in RV output and RV compression can compromise systemic cardiac output and coronary artery perfusion. RV ballooning will also result in tricuspid regurgitation and elevated systemic venous pressures, compromising perfusion pressures of end-organs.

During PARDS, the developing heart of a neonate or young infant is rather suddenly exposed to a potentially toxic cardiorespiratory milieu. Intrathoracic positioning intrinsically subjects the right atrium and RV to transpulmonary pressures changes. During positive pressure ventilation with high mean airway pressures, right atrial filling is limited due to a reduction in transmural pressure, which may decrease RV preload (though this effect may be diminished as lung compliance worsens). While positive pressure ventilation generally reduces transmural pressures across both ventricles (which would be expected to reduce ventricular afterload and metabolic demand), increases in PVR observed during PARDS can overwhelm the modest reduction in RV afterload and stress the RV myocardium. As PARDS severity worsens, PVR rises due to hypoxia-/hypercarbia-mediated vasoconstriction, reduction in recruited alveolar units, and higher mean airway pressures generated in an effort to sustain systemic oxygenation. These stressors to the RV are not easily correctable. The RV of an older child generally has the capacity to handle acute increases in RVEDP and RVEDV by mounting an increase in RV contractility and moderate dilatation (Figure 2). As discussed above, the neonatal myocardium has much less ability to respond to acute changes in volume and pressure experienced during severe PARDS.

### RV dysfunction: definition, incidence, and evaluation

To the credit of the PALICC investigators, the RV is mentioned as a potential culprit worthy of interrogation in the setting of “suspected cardiac dysfunction” during PARDS (10). Unfortunately, absence of a formal definition for RV dysfunction in children precludes uniform diagnosis or management. Even in adult practice, precise definitions for RV dysfunction and RV failure remain elusive. These pathophysiologic manifestations may be more practically distinguished by the RV myocardial response to increased RVEDV, as offered by Vieillard-Baron et al. (23) (Figure 2). In states of RV dysfunction, though myocardial contractility may be impaired, the ventricle is able mount a response to higher preload by increasing, or at least maintaining, stroke volume without developing systemic venous congestion. As RV systolic function deteriorates, the RV is unable to increase or maintain stroke volume in response to incremental increases in RVEDV, leading to impaired RV outflow. In this state of RV failure, reduced RV output cascades into lower LV preload; systemic-to-suprasystemic RVEDP with resultant RV ballooning, LV compression, and tricuspid regurgitation; and ultimately systemic venous congestion with reduced end-organ perfusion.

While a general gestalt exists amongst pediatric cardiologists and intensive care providers, an objective, operational definition of pediatric RV dysfunction or failure remains elusive for a number of important reasons. First, there is not a consensus among pediatric cardiologists regarding whether RV dysfunction should be categorized according to systolic dysfunction, diastolic dysfunction, or both. Second, while most pediatric cardiologists and pediatric intensive care providers would agree that a battery of biomarkers and echocardiographic data are essential to diagnosing RV dysfunction at the bedside, the precise assays and ultrasonic readouts required for diagnosis continue to be debated. Moreover, many echocardiographic measures commonly employed to determine RV function in adults remain unvalidated in children and are rarely performed by pediatric sonographers.

#### Epidemiology

Early and persistent RV hypertension and dysfunction have been associated with higher mortality in children with ARDS (24, 25). However, as uniform definitions for RV dysfunction, RV failure, and ACP remain unclear at this time, it is difficult to accurately report the incidence of or risk factors for PARDS-associated RV dysfunction. Therefore, we must again lean on literature from adult ARDS populations to begin understanding the prevalence of this disease. Two separate prospective cohort studies report incidences of the severest form of RV failure, acute cor pulmonale (ACP), to be nearly 20% during moderate-to-severe ARDS managed with protective lung ventilation (26, 27). Thus, it is reasonable to postulate that RV dysfunction is present in nearly 1 out of every 4 children with moderate-to-severe PARDS and that RV failure is an important driver of PARDS-associated mortality.

In adults with ARDS, risk factors for developing ACP include pneumonia as the etiology for ARDS, PaO_2_/F_I_O_2_ ratio <150 mmHg, PaCO_2_ ≥48 mmHg, and driving pressure ≥18 cmH_2_O (27). Though further research is warranted to characterize discrete risk factors for RV dysfunction and failure in PARDS, given a recent series of RV failure in infants with bronchopulmonary dysplasia admitted to our institution for PARDS, PICU providers at our center have adopted a policy of greater vigilance for RV dysfunction in children with underlying cardiopulmonary disease (e.g., congenital heart disease, bronchopulmonary dysplasia, sickle cell anemia, chronic kidney disease with long-standing hypertension) who develop severe PARDS. Moreover, our institute has adopted a policy in which development of multiorgan failure during PARDS immediately prompts interrogation of RV function as a potential cause of end-organ damage. In light of the paucity of evidence around the incidence and risk factors for PARDS-associated RV dysfunction, it is imperative that the American Society of Echocardiography and European Association of Cardiovascular Imaging settle on a formal definition of RV dysfunction for PARDS epidemiologic reporting purposes and for future PARDS research initiatives.

#### Clinical signs and biomarkers

In the absence of consensus recommendations, many pediatric centers rely upon complementary exam findings, noninvasive and invasive monitoring readouts, and biomarker data, in concert with echocardiographic measures of RV performance, to identify RV dysfunction. Clinical exam findings that might suggest RV dysfunction during PARDS include persistent atrial tachycardia in the presence of preserved LV systolic function, rising central venous pressure (CVP), and inspiratory pulse pressure variation (reverse pulsus paradoxus) on invasive arterial pressure monitoring that is unresponsive to volume expansion. Children with RV dysfunction commonly develop acute hepatomegaly that can result in abdominal distention and worsening respiratory system compliance from thoraco-abdominal competition. Anasarca that is unrelated to fluid overload manifests due to a persistently elevated CVP that leads to elevated hydrostatic pressures in peripheral microvascular beds. Similarly, high CVP may reduce lymphatic drainage into the subclavian veins that can result in pleural effusions and ascites. Feeding intolerance manifest as a reduction in intestinal perfusion pressure and due to the peritoneal space occupation by severe hepatomegaly. Elevated plasma levels of brain-type natriuretic peptide (BNP) or N-terminal pro-BNP are sensitive but nonspecific markers of RV wall stretch and, in the absence of frank renal failure, are commonly employed to evaluate for RV wall stress and right atrium/RV dilation (28–30). Climbing plasma levels of direct bilirubin and creatinine may point to decompensation in liver or renal perfusion pressures, respectively, as a result of elevated right-sided heart pressures with back-filling of the vena cavae. Recognition of any of these clinical markers in a child with PARDS should prompt echocardiographic assessment of RV size and function.

#### Ultrasound evaluation

Ultrasonographic assessment, both by formal comprehensive echocardiography and by point-of-care ultrasound, is now one of the hallmark methods for diagnosing RV dysfunction in PARDS. In the PICU, transthoracic echocardiography (TTE) is most commonly employed because of its noninvasive approach and capability of being performed at the bedside by a sonographer without the express need for a cardiologist present. TTE also affords the ability for serial examination of the heart in concert with dynamic changes during the course of PARDS. However, adequate acoustic windows can be challenging to acquire during PARDS, and the retrosternal position of the RV may further complicate proper image acquisition. Table 2 summarizes reported echocardiographic readouts of RV performance and their advantages/disadvantages.

**Table 2:** 2D echocardiographic measures of right ventricular function during PARDS (31, 32).

Metric	Functional measure	Strength	Weakness
*Dimension changes*			
RA diameter	Diastole	Commonly evaluated	Less helpful without prior echocardiographic measurement Preload-dependent
IVC size, respiratory variation	Diastole	Same as above	Subjective Preload-dependent
Interventricular septal position	Diastole	Same as above	Same as above
RV-to-LV end-systolic diameter ratio	Diastole	Easy to measure	Less commonly evaluated in children Difficult to visualize lateral wall May poorly predict RV volume changes
TAPSE	Systole	May correlate with RV ejection fraction Easy to measure Quantitative read-out	Less commonly evaluated in children Preload-dependent *Z* scores unvalidated in children
Fractional area change^a^	Systole	Improvement over linear dimension changes Quantitative read-out Adult data available	Preload-dependent Difficult to visualize lateral wall Unvalidated in children
*Wall strain pattern*			
2D speckle tracking	Systole	Less preload-dependent	Dependent on probe alignment High noise-to-signal ratio Requires additional software and complex analysis Unvalidated in children
*Doppler*			
Tricuspid insufficiency peak velocity (*V*_TI_)^b^	Systole	Commonly evaluated Quantitative read-out	Dependent on probe alignment Less commonly evaluated in children Preload-dependent Reliant on tricuspid insufficiency
d*P*/d*t*^c^	Systole	Quantitative read-out	Same as above
Myocardial performance index^d^	Systole, diastole	Quantitative read-out Adult data available	Dependent on probe alignment Less commonly evaluated in children Preload-dependent
Isovolumetric acceleration	Systole	Quantitative read-out	Same as above
Tissue doppler imaging	s: systole e′, a′: diastole	Easy to measure Pediatric data available	Same as above

2D, two-dimensional; d*P*/d*t*, rate of rise of intraventricular pressure during isovolumetric contraction; IVC, inferior vena cava; LV, left ventricle; RA, right atrium; RV, right ventricle; TAPSE, tricuspid annular plane systolic excursion.

a Fractional area change of RV is determined by the formula: (end-diastolic area − end-systolic area)/end-diastolic area.

b *V*_TI_ is used to approximate peak RV systolic pressure by the formula: average right atrial pressure + 4*(*V*_TI_)^2^.

c d*P*/d*t* (mmHg*sec^−1^) is determined by measuring the time (milliseconds) required to progress from an initial velocity (*V*_1_, m*sec^−1^; typically 1 m*sec^−1^ is used) to a second velocity (*V*_2_, m*sec^−1^; typically 2 m*sec^−1^ is used) tricuspid insufficiency envelope detected by continuous-wave doppler. d*P*/d*t* is then calculated by the formula: [(4*V_2_^2^) − (4*V_1_^2^)]/(time*0.001). For example, if 25 milliseconds are required for a tricuspid insufficiency jet velocity profile to increase from 1 m*sec^−1^ to 2 m*sec^−1^, d*P*/d*t* is equal to [(4*2^2^) − (4*1^2^)]/(25*0.001), or 480 mmHg*sec^−1^.

d Myocardial performance index is determined by the formula: (time in isovolumetric contraction + time in isovolumetric relaxation)/duration of RV ejection.

The American Society of Echocardiography recommends both qualitative and at least one quantitative assessment to determine RV systolic function in adults (31). Though the American Society of Echocardiography offer various quantification protocols for evaluating pediatric RV function, formal recommendations are less well developed (32, 33). RV performance can be measured by ventricular dimension changes, wall strain pattern, and doppler readouts throughout the cardiac cycle.

Due to its complex geometry, singular measurements of linear dimensions are rarely sufficient functional readouts, and commonly used measures of LV function (e.g., shortening fraction, ejection fraction) are unreliable with two-dimensional sonography of the RV. Qualitatively, using an apical 2 or 4 chamber view, RV diameter can be grossly, though subjectively, compared to LV diameter. This assessment may be further quantified as the RV/LV end-systolic diameter ratio. In older children (average age ~8.5 years), normal RV/LV ratios have been reported to be <0.6 with values >1 being associated RV hypertension and adverse clinical outcomes (34). Qualitative assessment of the interventricular septum can be performed in the parasternal short axis: flattening of the septum and a D-shaped LV in this view are indicative of RV hypertension and elevated RVEDV. Enlargement of the right atrium and inferior vena cava with limited respiratory variation in the diameter of the inferior vena cava can point to elevated RVEDP, resulting in limited RV filling and venous congestion. However, these measures provide only a vague estimation of elevated RV pressures. Furthermore, as pulmonary arteriolar resistance may require 6–12 months to reduce to normal adult-like physiology following birth, further work is needed to generate validated ratios in neonates and infants.

To begin quantitative assessment of RV function, pediatric cardiologists commonly measure peak velocity of the tricuspid insufficiency jet during systole (*V*_TI_) using continuous-wave doppler. When combined with an average CVP measured in the superior vena cava or right atrium, this peak velocity can provide a more objective measure of systolic pressures the RV is capable of mounting [RV systolic pressure = average right atrial pressure + 4*(*V*_TI_)^2^]. Longitudinal shortening can be measured quantitatively using tricuspid annular plane systolic excursion (TAPSE) (35). TAPSE is best evaluated in an apical 2 or 4 chamber view employing M mode to track the distance moved by the lateral tricuspid valve annulus in a single cardiac cycle. In adults, TAPSE ≥18 mm is considered normal while <17 mm is highly suggestive of systolic dysfunction (36). There are TAPSE values indexed to body surface area that can be referenced for pediatric patients (37); however, this readout is infrequently measured and reported in pediatric echocardiography and specific *Z* score values for children remain unvalidated.

Fractional area change of the RV can also provide a quantitative measure of systolic function. In the apical view, the RV cavity is traced and measured in end-diastole and end-systole. A reduction of at least 1/3 of the area from diastole to systole suggests normal RV systolic function in adults (38). However, validated, normal values for children are unknown. Moreover, accuracy of the RV fractional area change to predict RV systolic function diminishes as RVEDV increases (39).

RV wall strain pattern has become a helpful adjunct to diagnose RV systolic dysfunction in adults (40, 41). A recent report by Romanowicz et al. (42) provides validated RV strain values and *Z* scores for children using two-dimensional speckle tracking echocardiography, which may prove helpful in quantifying RV systolic function during PARDS in the years to come. However, until RV strain measures are more consistently performed by pediatric cardiologists and reported strain values are more widely circulated in literature, the utility of employing RV strain values to diagnosis new or persistent RV systolic dysfunction during PARDS remains unknown.

In addition to changes in RV geometric dimensions or RV wall strain, various doppler measurements of RV performance may have utility in diagnosing RV systolic and/or diastolic dysfunction during PARDS. Tissue doppler imaging of the RV free wall at the level of the tricuspid annulus can be used to measure myocardial systolic peak velocity (s′), early diastolic velocity (e′), and late diastolic velocity (a′); together, these readouts provide valuable real-time information on RV myocardial contractility and relaxation. To corroborate RV s’ data and detail RV systolic function, sonographers may capture the isovolumetric acceleration of the basilar aspect of the RV free wall that can provide more specific information on RV longitudinal shortening. Myocardial performance index (Tei index), measured as the sum of the duration of the cardiac cycle in RV isovolumetric contraction and relaxation divided by the duration of RV ejection, may provide a sensitive readout suggestive of either RV systolic or diastolic dysfunction. Finally, in the presence of tricuspid insufficiency, changes in tricuspid regurgitation velocity can be measured and reported as a change in pressure over change in time (d*P*/d*t*), approximating the rate of rise in RV pressure during early systole reflective of RV systolic performance. Though each of these advanced imaging modalities may eventually prove useful (or even essential) in diagnosing RV dysfunction in the setting of PARDS in the future, their availability and scope of use outside the management of congenital heart disease remain quite limited.

## Therapeutic strategies for RV dysfunction during PARDS

Little evidence can be amassed to guide the treatment for PARDS-mediated RV dysfunction, leaving the pediatric intensivist to rely on evidence for treating RV dysfunction in the setting of other etiologies, extrapolation from literature in adult populations, and/or on basic understanding of cardiopulmonary pathophysiology during PARDS. Here we will summarize the most frequently used therapeutic strategies for treating RV dysfunction during PARDS and their supporting evidences (Table 3), focusing on (1) reduction of RV afterload, (2) restoration and sustenance of RV contractility, and (3) optimization of RV diastolic function. However, the prioritization and urgency for implementing these RV protective strategies remains unknown at this time.

**Table 3:** Proposed therapies for right ventricular dysfunction in pediatric acute respiratory distress syndrome.

Therapy	Purpose and mechanism	References
*MV strategies*	￬ PVR by:	
Limit driving pressure	↓ RV afterload	
Titrate mean airway pressure	Restore FRC, ↑ PaO_2_ ➔ pulmonary vasodilation	
↑ F_I_O_2_	↑ PaO_2_ ➔ pulmonary vasodilation	
↑ Minute ventilation	↓ PaCO_2_ ➔ pulmonary vasodilation	
*Prone posture*	￬ PVR and RV afterload by:	(43–45)
	↓ Ventral-to-dorsal transpulmonary pressure gradient ➔ ↑ alveolar V/Q matching	
	↑ Respiratory system compliance ➔ ↓ driving pressure	
	Optimizing RV geometry	
*Pulmonary vasodilators*	￬ RV afterload by ￬ PVR	(46–48)
Inhaled nitric oxide	Guanylate cyclase activator	
Inhaled epoprostenol, iloprost	PGI_2_-receptor agonist	
Milrinone	PA PDE-3 inhibitor	
*Inotropic agents*	Increase RV, LV contractility	(46, 49)
Epinephrine, dobutamine	Myocardial β_1_-receptor agonist	
Milrinone Ca^2+^ (neonates)	Myocardial PDE-3 inhibitor Bind troponin C, exposing myosin binding sites on actin	
*Vasoactive agents*	Increase SVR and coronary perfusion pressure	(46, 50, 51)
Norepinephrine	Systemic arteriolar α_1_-receptor agonist	
Vasopressin	Systemic arteriolar V_1_-receptor agonist May ￪ NO in PAs and thus ￬ PVR	
*Fluid management*	Judiciously ￬ RVEDP by ￬ RVEDV	(52, 53)
Loop, thiazide diuretics	Sodium and free water excretion	
CRRT	Plasma ultrafiltration	
*ECMO*	Rescue therapy	
VV VA/VP	￬ PCO_2_ and ￪ pH, PO_2_ in PAs ￬ RV preload and thus ￬ RVEDP Maintain systemic oxygen delivery in a failing RV	

cAMP, cyclic adenosine monophosphate; CRRT, continuous renal replacement therapy; ECMO, extracorporeal membrane oxygenation; FRC, functional residual capacity; MV, mechanical ventilation; NO, nitric oxide; PA, pulmonary artery; PCO_2_, partial pressure of carbon dioxide; PDE-3, phosphodiesterase 3; PO_2_, partial pressure of oxygen; PVR, pulmonary vascular resistance; RV, right ventricle; RVEDP, right ventricular end-diastolic pressure; SVR, systemic vascular resistance; VA, venoarterial; VP, veno-pulmonary arterial; V/Q, ventilation/perfusion; VV, venovenous.

### Mechanical ventilation management

There is little evidence to guide the management of invasive ventilation when RV dysfunction is suspected or confirmed in a child with PARDS beyond PALICC recommendations. Informed by clinical data from ARDS studies in adults (3), it is reasonable to employ the following strategies to reduce PVR and RV afterload. As oxygen is a selective pulmonary vasodilator, F_I_O_2_ may be temporarily increased and mean airway pressure judiciously titrated to optimize dynamic compliance or respiratory system impedance (assuming negligible contribution of airway resistance to the patient’s respiratory mechanics) in an effort to preserve FRC (thus maximizing alveolar recruitment while limiting regional dead-space) and drive oxyhemoglobin saturation above 90%. Caution is warranted here as prolonged exposure to high F_I_O_2_ may contribute to ventilator-induced lung injury, and exposure to high mean airway pressure without re-evaluation of RV performance may precipitate further RV decline. Permissive hypercapnia goals should be tightened, though a precise upper limit of PaCO_2_ is unclear at this time. Acidemia should be corrected either with judicious increases in minute ventilation (while limiting driving pressure) and/or with increased circulating levels of bicarbonate. There is insufficient evidence to support mode of ventilation (e.g., conventional mechanical ventilation, high frequency oscillatory ventilation, airway pressure-release ventilation, etc.) to accomplish these goals, regardless of RV function (54). At present, it is unknown whether spontaneous respiratory effort is more advantageous for a dysfunctional RV during PARDS than neuromuscular blockade.

### Prone posture

Prone positioning as a treatment for ARDS and a tool to decrease ventilator-induced lung injury is well-described in adults (55–58). It has also been shown to have a role in unloading the RV and improving right ventriculo-arterial coupling (43). Proning can decrease PVR and increase cardiac output among adult patients with ARDS (44). Vieillard-Baron et al. (45) demonstrated an increase in cardiac index with 18 h of prone positioning of patients with ARDS-induced ACP. Moreover, an adult cohort with severe ARDS randomized to prone posture experienced a significantly lower incidence of cardiac arrest relative to those who remained supine (55). There are several proposed mechanisms for the hemodynamic benefit of prone positioning that culminate in reduced RV afterload (43). Despite increasing dorsal transpulmonary pressure (as measured by esophageal manometry) (59), the prone posture may increase overall respiratory system compliance by reducing the ventral-to-dorsal transpulmonary pressure differential (60). This improvement in respiratory mechanics reduces driving pressures while increasing the homogeneity of alveolar ventilation (43), limiting risk of ventilator-induced lung injury and associated RV dysfunction (61, 62). Improvements in ventilation and oxygenation associated with more optimal ventilation/perfusion matching would be expected to reduce PVR. Moreover, greater homogeneity in lung aeration would be expected to improve FRC toward baseline. Even modest restoration in FRC could reduce PVR and thus decrease RV afterload. There is also speculation that proning optimizes RV three-dimensional geometry, leading to improved RV systolic function (45). Though the evidence and mechanisms by which prone positioning promotes reductions in RV stress during ARDS have only been demonstrated in adults (summarized nicely by Vieillard-Baron et al. (43)), in the absence of evidence in the pediatric population, prone positioning can be reasonably employed for children with or at risk for acute RV dysfunction in the setting of PARDS.

### Pulmonary vasodilators

Much like the goal of reducing systemic vascular resistance (SVR) in the setting of LV systolic failure, therapies that directly target PVR reduction are logical to prescribe in the setting of PARDS-related RV systolic dysfunction. The most commonly prescribed first-line treatment for increased PVR during PARDS is inhaled nitric oxide (iNO) (46) given its demonstrable reduction of PVR in adults with ARDS (63). Mechanistically, iNO induces pulmonary arterial vasodilation in regions of well-ventilated lung by stimulating guanylate cyclase in pulmonary arteriolar smooth muscle cells to generate cyclic guanosine monophosphate (64). However, despite evidence that iNO improves oxygenation during PARDS, children do not perceive a survival benefit with its use (65). Though other inhaled pulmonary vasodilators [e.g., inhaled epoprostenol (or prostacyclin) and iloprost (a synthetic analog of prostacyclin)] may have similar effects on PVR and systemic oxygenation as iNO, these therapies are intensely potent and may result in rebound pulmonary arterial hypertension if inadvertently discontinued. Therefore, their use in the PICU remains restricted to the management of isolated pulmonary hypertension without coincident PARDS (46–48).

Orally administered systemic vasodilators, such as sildenafil (phosphodiesterase 5 inhibitor), ambrisentan (endothelin receptor antagonist), bosentan (endothelin receptor antagonist), and riociguat (stimulator of guanylate cyclase), are typically utilized in children with chronically elevated PVR from diseases such as bronchopulmonary dysplasia, congenital heart disease, or sickle cell anemia (47, 66). In children with known pulmonary disease who present to the PICU with severe PARDS, critical care providers commonly continue these home therapies even in the absence of RV dysfunction for the hemodynamically stable child. Alternatively, in the previously healthy child now presenting with severe PARDS, our experience suggests that these therapies are not commonly considered unless the patient manifests persistent RV systolic dysfunction during a prolonged course of PARDS. In the face of a dearth of evidence to guide the use of inhaled or systemic vasodilators to treat or prevent RV systolic dysfunction during PARDS, these therapies cannot be universally recommended in all children with severe PARDS (10).

### Inotropic and vasoactive agents

In the absence of robust evidence to support the use of inotropic or vasoactive agents for RV systolic dysfunction with PARDS, it is logical to reach for these therapies to sustain RV contractility and the LV contribution to RV output in an effort to maintain forward pulmonary blood flow and prevent RV bowing into the LV cavity. Low dose epinephrine (<0.05 μg/kg/min) or dobutamine (1–20 μg/kg/min) are catecholamines used to promote inotropy through β_1_ G-protein coupled receptors with variable activity on pulmonary arterial vasodilatation through β_2_-receptors (50, 67). The benefit of epinephrine and dobutamine over other inotropes (e.g., digoxin, milrinone) lies in their capacity for rapid titration. However, both catecholaminergic agents improve inotropy at the cost of increased myocardial oxygen demand.

Calcium is an important inotrope in an infant with RV dysfunction. Prior work has demonstrated that infants treated with intravenous calcium following cardiopulmonary bypass demonstrated significant improvements in cardiac output and mean systemic blood pressure compared to infants who did not receive calcium (68). Similarly, multiple case series attest to the incidence of myocardial dysfunction in infants manifesting nutrition-mediated hypocalcemia (69, 70). Physiologically, these findings may be explained by the underdeveloped t-tubular system of the myocardial sarcomere and an underdeveloped sarcoplasmic endoreticulum (evidence of which has been helpfully summarized by Baum and Palmisano (17)). The developmental immaturity of these myocardial structures demand that sarcomeric contractility in an infant’s heart relies heavily on extracellular ionized calcium availability to facilitate myosin-actin interactions. Therefore, ensuring normal circulating levels of ionized calcium in young children may be necessary to sustain RV systolic function during PARDS. In a similar way, it may be logical to leverage the calcium-sensitizing effects of levosimendan to sustain or improve RV systolic function in a child with PARDS. However, the quality of evidence for the use of levosimendan in children with primary cardiac disease remains poor (71), and the evidence for levosimendan use outside of acute-on-chronic heart failure in children or adults is wholly lacking.

Milrinone may be considered in a hemodynamically stable children RV dysfunction due to PARDS. Milrinone is a phosphodiesterase 3 inhibitor, promoting pulmonary and systemic vasodilation along with myocardial inotropy by decreasing the degradation of cyclic adenosine monophosphate within vascular smooth muscle and myocardium, respectively. However, milrinone should be used with caution. It may promote global pulmonary vasodilatation that could worsen the respiratory shunt fraction and exacerbate systemic hypoxemia. Moreover, the long half-life and renal clearance of milrinone may precipitously lead to refractory hypotension in a child developing impaired renal function (72). In a hemodynamically unstable patient, milrinone is best used in conjunction with a vasopressor if used at all (49). In the setting of ACP, decreased SVR caused by milrinone could theoretically reduce LV end-diastolic pressure and paradoxically worsen LV compression by the ballooning RV (46).

Norepinephrine has been suggested in experimental models to improve RV function and cardiac output (4). By increasing SVR through the activation of α_1_-receptors, norepinephrine raises the systemic diastolic pressure and thus may improve coronary perfusion. Norepinephrine also has mild activity on myocardial β_1_-receptors that can improve both RV contractility and the LV contribution to RV cardiac output. In addition, norepinephrine increases LV afterload that results in higher LV end-diastolic pressures to compete against the rightward-shifting interventricular septum during RV failure (73). However, as with the use of epinephrine, care must be taken to recognize that norepinephrine will increase myocardial oxygen demand in an already stressed heart. Vasopressin, on the other hand, may be used in the hemodynamically unstable child with PARDS-mediated RV systolic dysfunction to maintain systemic arterial pressures without directly increasing myocardial oxygen demand. Vasopressin raises SVR by activating V_1_-receptors in systemic arterioles, thus promoting increased intracellular calcium availability through the activation of phospholipase C. Importantly, vasopressin has a smaller effect on PVR than SVR (74). The mechanism is thought to be due to V_1_-receptor-mediated nitric oxide release in the pulmonary vasculature that leads to vasodilation (51). Vasopressin has been shown to consistently decrease the pulmonary-to-aortic systolic pressure ratio in pediatric patients with known pulmonary hypertension (50). These properties suggest vasopressin as an ideal vasopressor choice in the setting of hemodynamically unstable RV systolic failure (46).

### Fluid management

Fluid management during PARDS is a complex task. The conservative approach of fluid restriction and/or diuresis may reduce extravascular lung water and thus improve ventilation/perfusion matching; however, this may come at the cost of reduced intravascular volume and end-organ perfusion. Current recommendations for fluid management in the setting of PARDS focus on goal-directed care (neither conservative nor liberal in approach) (75). Typically, a child with normal biventricular function handles the significant changes in intravascular volume that occur between initial volume loading during resuscitation of shock and the aggressive diuresis that commonly follows cardiovascular stabilization in the setting of PARDS. However, RV function is particularly sensitive to the complex cardiopulmonary changes that occur with intravascular fluid shifts, especially during positive pressure ventilation with high mean airway pressure. An accurate assessment of intra- and extravascular volume status of a child in acute RV dysfunction is thus critical for preserving and/or restoring RV performance during PARDS.

The notion of preload dependence in a failing RV has merit, and increasing intravascular volume in an acutely hemodynamically unstable patient may be necessary. However, excess preload can worsen RV dilatation, resulting in septal bowing into the LV, tricuspid regurgitation with worsening venous congestion, and increased RV myocardial wall tension that compromises coronary perfusion pressure and may precipitate clinical decompensation (18, 52, 76–78). Though pulmonary arterial catheters are used exceedingly rarely in pediatrics, invasive monitoring using CVP trends can be helpful to guide the need for decongestion and to better understand the right atrial pressure necessary to provide adequate preload (53). Decongestion allows for decompression of the RV, reducing ventricular interdependence, and improving hemodynamics overall (49). Decongestion is primarily achieved through judicious diuresis, typically with the use of intravenous loop diuretics (e.g., furosemide, bumetanide) with or without the use of thiazide diuretics (e.g., intravenous chlorothiazide or, in children with sustained gut function, metolazone). There are many barriers to effective diuresis in patients with right heart dysfunction including acute kidney injury mediated by high CVP, low cardiac output, and the resulting reduction in renal perfusion pressure (79). In a hypervolemic patient with acute RV dysfunction, vasopressors may be required to sustain sufficient renal perfusion pressure while diuretic therapy is use to achieve RV decompression and venous decongestion (49). For children who fail to respond to diuretic therapy, continuous renal replacement therapy (CRRT) may be necessary to achieve intravascular volume removal (80). However, the need for CRRT in the setting of PARDS-associated RV dysfunction should heighten the pediatric intensivist’s alertness to the patient’s manifestation of extremis and impending cardiovascular collapse. In such a clinical situation, the ethical considerations and risks for deploying CRRT must be strongly weighed against any perceived benefit to the patient.

### Extracorporeal membrane oxygenation

The decision to deploy extracorporeal membrane oxygenation (ECMO) for severe PARDS refractory to lung-protective ventilation is often challenging and emotionally charged. The pediatric intensivist often has to guide a family through the complex risks and benefits of deploying ECMO for their child in a time-sensitive manner with limited information. When RV dysfunction develops in the setting of severe PARDS, complex treatment decisions can become substantially more intricate. Here we would like to discuss (1) the decision to use venovenous (VV) versus venoarterial (VA) ECMO as the initial cannulation strategy for PARDS complicated by RV dysfunction and (2) specific treatment decisions around RV dysfunction during VV ECMO.

#### VV versus VA ECMO

Peripheral VV or VA ECMO are the predominant support modalities used to support children with severe PARDS (81). However, Extracorporeal Life Support Organization guidelines are not clear as to the preferential approach for children with concomitant RV dysfunction (82). Theoretically, RV dysfunction does not immediately preclude VV ECMO given its capability of normalizing pH and PaCO_2_ and restoring precapillary oxygenation may reduce PVR and improve RV systolic function (83–86). Indeed, reductions in pulmonary arterial and central venous pressures, as well as increases in cardiac index, have been seen with initiation of VV ECMO without adjustments to mechanical ventilation or vasopressor/inotropic support (27, 84, 87–89). VV ECMO also has the benefit of not invading peripheral arteries, which may reduce the risk of bleeding and neurologic complications when compared to VA ECMO deployment through neck vessels (90). On the other hand, VV ECMO does not significantly reduce RV preload and carries the risk of recirculation. Moreover, should RV systolic function continue to deteriorate, VV ECMO can do nothing to provide systemic oxygen delivery and instead results in greater recirculation.

VA ECMO is more commonly selected for children with PARDS in whom VV cannulation is technically not feasible or cardiac failure is also present. VA ECMO through the right internal jugular vein and right common carotid artery diverts systemic venous return from the right atrium to a membrane oxygenator for eventual return to the arterial system distal to the aortic valve. In so doing, preload to the RV and pulmonary vasculature decreases, which would be predicted to reduce RV wall stress, RV afterload, and RV myocardial oxygen demand. It is important to note that carotid return of ECMO blood flow may increase LV afterload and shift myocardial stress from the RV to the LV (91). However, in our experience, most children with severe PARDS requiring ECMO have well-preserved LV function that can withstand the increased afterload. Despite VA ECMO having clear physiological advantages over VV ECMO for children with PARDS and RV dysfunction, neurological risks and overall goals of care must be weighted heavily by all providers in the ECMO cannulation process. Furthermore, it is reasonable, where feasible, to consider transition from VA to VV ECMO in a patient in whom cardiac failure has sufficiently resolved but persistent severe PARDS precludes the sustainability of lung-protective ventilation without ongoing extracorporeal support.

#### VV ECMO-specific considerations

One of the main goals in using VV ECMO for severe PARDS is to reduce ventilator-induced lung injury while supporting systemic oxygen delivery and carbon dioxide removal. Though optimal ventilator support during VV ECMO is presently unclear, lung protective strategies remain the mainstay of respiratory management during ECMO. During the process of weaning ventilator settings following ECMO deployment, lung de-recruitment is commonplace, potentially worsening RV afterload. Maintaining “adequate PEEP” while on ECMO has been shown to improve survival (92, 93); however, the precise definition of “adequate PEEP” during pediatric ECMO remains unclear. The use of VV ECMO, in particular, may not adequately support the RV in spite of its theoretical benefits of optimizing pulmonary microvascular pH, PCO_2_, and PO_2_.

If undiagnosed on pre-ECMO evaluation, the development of RV dysfunction is commonly insidious during VV ECMO and may portend cardiopulmonary collapse (94). Therefore, pediatric intensivists must maintain a high index of suspicion for RV dysfunction throughout the ECMO run. Currently there are no guidelines to inform how and when to evaluate for RV dysfunction during VV ECMO. However, as described above, serial evaluation of the clinical exam, circulating biomarkers of end-organ function, and echocardiographic measures of RV performance can be leveraged to identify RV dysfunction early (4, 95, 96). Early identification of RV dysfunction is critical as evidence of RV dilatation and abnormal septal movement post-cannulation are associated with failure to wean from ECMO and increased mortality (94, 97, 98).

It is unknown whether therapies employed prophylactically to reduce RV stress (e.g., iNO, milrinone, prone positioning (27, 99, 100), diuresis (4, 86, 101, 102)) are helpful to mitigate the risk of acquiring RV dysfunction during VV ECMO. Furthermore, when RV dysfunction is uncovered, it is unclear whether conversion to VA ECMO, or the more recently described veno-pulmonary arterial ECMO (103), before RV failure is present can facilitate RV recovery. Once RV failure fully manifests, however, conversion to VA ECMO or implementation of an RV assist device is typically required to salvage the patient as precipitous cardiac arrest is often soon to follow.

We would like to highlight one final consideration in the management of pediatric VV ECMO germane to the patient with known RV dysfunction: use of β-blockers in the management of refractory hypoxemia. Such a clinical scenario is typically reached only when hypoxemia is clinically important (manifested by rising lactate or limitation in other goals of care such as wakefulness), the primary etiology is an isolated elevation in cardiac output, and other potential diagnoses are ruled out or treated. Bunge et al. (104) reported a case series of 33 adults treated with β-blockers for hypoxemia during VV ECMO without incidence of new or worsening RV dysfunction. Guarracino et al. (105) reported their experience in managing 3 adults with sepsis who developed hypoxemia during VV ECMO due to elevated cardiac output. In this small cohort, all patients demonstrated improved systemic oxygenation with an esmolol infusion, though echocardiographic and clinical outcome data were not reported. β-blockers are negative inotropes and thus can promote or exacerbate RV myocardial dysfunction. Therefore, in our estimation, these agents should be prescribed with caution during VV ECMO when RV dysfunction is absent and should be avoided when RV dysfunction is present.

## Conclusion

Although a precise definition for RV dysfunction in children has not been settled, it is clear that embarrassment of RV systolic and diastolic function in the setting of PARDS is associated with worse clinical outcomes. PARDS outcomes are usually not dictated by PARDS severity alone and appear to have a greater association with the development of multiorgan failure (2, 106). We postulate that an under-recognized but potentially significant driver of multiorgan failure during PARDS is RV dysfunction and eventual RV failure. The precise incidence of RV dysfunction during PARDS that may better delineate RV dysfunction as a risk factor for PARDS-associated outcomes is currently unknown. We wish to call attention to this insidious pathophysiology during PARDS as its incidence is likely higher than appreciated, and we encourage more a concerted effort by the pediatric critical care research community to help fill this knowledge gap. If RV dysfunction is a significant risk factor for PARDS-associated outcomes as we suspect, then it may behoove pediatric providers to surveil for RV dysfunction sooner and more frequently during the course of severe PARDS. However, additional knowledge gaps include whether early identification of RV dysfunction in the course of severe PARDS or whether aggressive intervention to prevent or attenuate RV dysfunction during PARDS will improve outcomes.

## Author contributions

LW, JL, PP, and RR equally contributed to the conception of the manuscript. LW, LB, AM, PP, JL, and RR substantially contributed to the writing and revision of the manuscript and approve its final draft. All authors contributed to the article and approved the submitted version.

## Funding

RR is funded through the National Institutes of Health National Institute of General Medical Sciences grant 5K08 GM144788-02. This grant was used to cover the publication cost for this manuscript.

## Conflict of interest

The authors declare that the research was conducted in the absence of any commercial or financial relationships that could be construed as a potential conflict of interest.

## Publisher’s note

All claims expressed in this article are solely those of the authors and do not necessarily represent those of their affiliated organizations, or those of the publisher, the editors and the reviewers. Any product that may be evaluated in this article, or claim that may be made by its manufacturer, is not guaranteed or endorsed by the publisher.

## Figures and Tables

**Table:** 

ACP	acute cor pulmonale
ARDS	acute respiratory distress syndrome
BNP	brain-type natriuretic peptide
CRRT	continuous renal replacement therapy
CVP	central venous pressure
ECMO	extracorporeal membrane oxygenation
F_I_O_2_	fraction of inspired oxygen
FRC	functional residual capacity
iNO	inhaled nitric oxide
LV	left ventricle
PALICC	Pediatric Acute Lung Injury Consensus Conference
PARDS	pediatric acute respiratory distress syndrome
PEEP	positive end-expiratory pressure
PICU	pediatric intensive care unit
PVR	pulmonary vascular resistance
RV	right ventricle
RVEDP	right ventricular end-diastolic pressure
RVEDV	right ventricular end-diastolic volume
SVR	systemic vascular resistance
TAPSE	tricuspid annular plane systolic excursion
TTE	transthoracic echocardiography
VA	venoarterial
*V*_TI_	peak velocity of the tricuspid insufficiency jet during systole
VV	venovenous

## References

[1] Yehya N Smith L Thomas NJ Steffen KM Zimmerman J Lee JH. Definition, incidence, and epidemiology of pediatric acute respiratory distress syndrome: from the second Pediatric Acute Lung Injury Consensus Conference. Pediatr Crit Care Med. (2023) 24:S87–98. doi: 10.1097/PCC.0000000000003161 36661438

[2] Dowell JC Parvathaneni K Thomas NJ Khemani RG Yehya N. Epidemiology of cause of death in pediatric acute respiratory distress syndrome. Crit Care Med. (2018) 46:1811–9. doi: 10.1097/CCM.0000000000003371 30095498 PMC6185780

[3] Ganeriwal S Alves Dos Anjos G Schleicher M Hockstein MA Tonelli AR Duggal A. Right ventricle-specific therapies in acute respiratory distress syndrome: a scoping review. Crit Care. (2023) 27:104. doi: 10.1186/s13054-023-04395-9 36907888 PMC10008150

[4] Petit M Jullien E Vieillard-Baron A. Right ventricular function in acute respiratory distress syndrome: impact on outcome, respiratory strategy and use of veno-venous extracorporeal membrane oxygenation. Front Physiol. (2021) 12:797252. doi: 10.3389/fphys.2021.797252 35095561 PMC8795709

[5] Brown TN Brogan TV. Right ventricular dysfunction in patients with acute respiratory distress syndrome receiving venovenous extracorporeal membrane oxygenation. Front Cardiovasc Med. (2023) 10:1027300. doi: 10.3389/fcvm.2023.1027300 37265572 PMC10229794

[6] Sato R Dugar S Cheungpasitporn W Schleicher M Collier P Vallabhajosyula S. The impact of right ventricular injury on the mortality in patients with acute respiratory distress syndrome: a systematic review and meta-analysis. Crit Care. (2021) 25:172. doi: 10.1186/s13054-021-03591-9 34020703 PMC8138512

[7] Bernard GR Artigas A Brigham KL Carlet J Falke K Hudson L. The American-European Consensus Conference on ARDS. Definitions, mechanisms, relevant outcomes, and clinical trial coordination. Am J Respir Crit Care Med. (1994) 149:818–24. doi: 10.1164/ajrccm.149.3.7509706 7509706

[8] Ranieri VM Rubenfeld GD Thompson BT Ferguson ND Caldwell E Fan E. Acute respiratory distress syndrome: the Berlin definition. JAMA. (2012) 307:2526–33. doi: 10.1001/jama.2012.5669 22797452

[9] Ferguson ND Fan E Camporota L Antonelli M Anzueto A Beale R. The Berlin definition of ARDS: an expanded rationale, justification, and supplementary material. Intensive Care Med. (2012) 38:1573–82. doi: 10.1007/s00134-012-2682-1 22926653

[10] Pediatric Acute Lung Injury Consensus Conference Group. Pediatric acute respiratory distress syndrome: consensus recommendations from the pediatric Acute Lung Injury Consensus Conference. Pediatr Crit Care Med. (2015) 16:428–39. doi: 10.1097/PCC.0000000000000350 25647235 PMC5253180

[11] Brower RG Matthay MA Morris A Schoenfeld D Thompson BT Wheeler A. Ventilation with lower tidal volumes as compared with traditional tidal volumes for acute lung injury and the acute respiratory distress syndrome. N Engl J Med. (2000) 342:1301–8. doi: 10.1056/NEJM200005043421801 10793162

[12] Emeriaud G Lopez-Fernandez YM Iyer NP Bembea MM Agulnik A Barbaro RP. Executive summary of the second international guidelines for the diagnosis and Management of Pediatric Acute Respiratory Distress Syndrome (PALICC-2). Pediatr Crit Care Med. (2023) 24:143–68. doi: 10.1097/PCC.0000000000003147 36661420 PMC9848214

[13] Fernandez A Modesto V Rimensberger PC Korang SK Iyer NP Cheifetz IM. Invasive ventilatory support in patients with pediatric acute respiratory distress syndrome: from the second Pediatric Acute Lung Injury Consensus Conference. Pediatr Crit Care Med. (2023) 24:S61–75. doi: 10.1097/PCC.0000000000003159 36661436

[14] Dahmer MK Yang G Zhang M Quasney MW Sapru A Weeks HM. Identification of phenotypes in paediatric patients with acute respiratory distress syndrome: a latent class analysis. Lancet Respir Med. (2022) 10:289–97. doi: 10.1016/S2213-2600(21)00382-9 34883088 PMC8897230

[15] Bhalla A Baudin F Takeuchi M Cruces P Second Pediatric Acute Lung Injury Consensus Conference (PALICC-2) of the Pediatric Acute Lung Injury and Sepsis Investigators (PALISI) Network. Monitoring in pediatric acute respiratory distress syndrome: from the second Pediatric Acute Lung Injury Consensus Conference. Pediatr Crit Care Med. (2023) 24:S112–23. doi: 10.1097/PCC.0000000000003163 36661440 PMC9980912

[16] Firpo C Hoffman JI Silverman NH. Evaluation of fetal heart dimensions from 12 weeks to term. Am J Cardiol. (2001) 87:594–600. doi: 10.1016/s0002-9149(00)01437-5 11230845

[17] Baum VC Palmisano BW. The immature heart and anesthesia. Anesthesiology. (1997) 87:1529–48. doi: 10.1097/00000542-199712000-00032 9416738

[18] Haddad F Hunt SA Rosenthal DN Murphy DJ. Right ventricular function in cardiovascular disease, part I: anatomy, physiology, aging, and functional assessment of the right ventricle. Circulation. (2008) 117:1436–48. doi: 10.1161/CIRCULATIONAHA.107.653576 18347220

[19] Singh S White FC Bloor CM. Myocardial morphometric characteristics in swine. Circ Res. (1981) 49:434–41. doi: 10.1161/01.res.49.2.434 7249279

[20] Yamaguchi S Harasawa H Li KS Zhu D Santamore WP. Comparative significance in systolic ventricular interaction. Cardiovasc Res. (1991) 25:774–83. doi: 10.1093/cvr/25.9.774 1799909

[21] Woulfe KC Walker LA. Physiology of the right ventricle across the lifespan. Front Physiol. (2021) 12:642284. doi: 10.3389/fphys.2021.642284 33737888 PMC7960651

[22] Brener MI Masoumi A Ng VG Tello K Bastos MB Cornwell WK III. Invasive right ventricular pressure-volume analysis: basic principles, clinical applications, and practical recommendations. Circ Heart Fail. (2022) 15:e009101. doi: 10.1161/CIRCHEARTFAILURE.121.009101 34963308 PMC8766922

[23] Vieillard-Baron A Naeije R Haddad F Bogaard HJ Bull TM Fletcher N. Diagnostic workup, etiologies and management of acute right ventricle failure: a state-of-the-art paper. Intensive Care Med. (2018) 44:774–90. doi: 10.1007/s00134-018-5172-2 29744563

[24] Himebauch AS Yehya N Wang Y Conlon T Kilbaugh TJ McGowan FX. Early right ventricular systolic dysfunction and pulmonary hypertension are associated with worse outcomes in pediatric acute respiratory distress syndrome. Crit Care Med. (2018) 46:e1055–62. doi: 10.1097/CCM.0000000000003358 30095502 PMC6185756

[25] Himebauch AS Yehya N Wang Y McGowan FX Mercer-Rosa L. New or persistent right ventricular systolic dysfunction is associated with worse outcomes in pediatric acute respiratory distress syndrome. Pediatr Crit Care Med. (2020) 21:e121–8. doi: 10.1097/PCC.0000000000002206 31851127 PMC11215761

[26] Boissier F Katsahian S Razazi K Thille AW Roche-Campo F Leon R. Prevalence and prognosis of cor pulmonale during protective ventilation for acute respiratory distress syndrome. Intensive Care Med. (2013) 39:1725–33. doi: 10.1007/s00134-013-2941-9 23673401

[27] Mekontso Dessap A Boissier F Charron C Begot E Repesse X Legras A. Acute cor pulmonale during protective ventilation for acute respiratory distress syndrome: prevalence, predictors, and clinical impact. Intensive Care Med. (2016) 42:862–70. doi: 10.1007/s00134-015-4141-2 26650055

[28] Auerbach SR Richmond ME Lamour JM Blume ED Addonizio LJ Shaddy RE. BNP levels predict outcome in pediatric heart failure patients: post hoc analysis of the pediatric carvedilol trial. Circ Heart Fail. (2010) 3:606–11. doi: 10.1161/CIRCHEARTFAILURE.109.906875 20573993

[29] Chang WT Shih JY Hong CS Lin YW Chen YC Ho CH. Right ventricular expression of NT-proBNP adds predictive value to reveal score in patients with pulmonary arterial hypertension. ESC Heart Fail. (2021) 8:3082–92. doi: 10.1002/ehf2.13410 33955184 PMC8318442

[30] Cantinotti M. B-type cardiac natriuretic peptides in the neonatal and pediatric intensive care units. J Pediatr Intensive Care. (2016) 5:189–97. doi: 10.1055/s-0036-1583543 31110904 PMC6512419

[31] Rudski LG Lai WW Afilalo J Hua L Handschumacher MD Chandrasekaran K. Guidelines for the echocardiographic assessment of the right heart in adults: a report from the American Society of Echocardiography endorsed by the European Association of Echocardiography, a registered branch of the European Society of Cardiology, and the Canadian Society of Echocardiography. J Am Soc Echocardiogr. (2010) 23:685–713. doi: 10.1016/j.echo.2010.05.010 20620859

[32] Lopez L Colan SD Frommelt PC Ensing GJ Kendall K Younoszai AK. Recommendations for quantification methods during the performance of a pediatric echocardiogram: a report from the Pediatric Measurements Writing Group of the American Society of Echocardiography Pediatric and Congenital Heart Disease Council. J Am Soc Echocardiogr. (2010) 23:465–95. doi: 10.1016/j.echo.2010.03.019 20451803

[33] Lai WW Geva T Shirali GS Frommelt PC Humes RA Brook MM. Guidelines and standards for performance of a pediatric echocardiogram: a report from the task force of the Pediatric Council of the American Society of Echocardiography. J Am Soc Echocardiogr. (2006) 19:1413–30. doi: 10.1016/j.echo.2006.09.001 17138024

[34] Jone PN Hinzman J Wagner BD Ivy DD Younoszai A. Right ventricular to left ventricular diameter ratio at end-systole in evaluating outcomes in children with pulmonary hypertension. J Am Soc Echocardiogr. (2014) 27:172–8. doi: 10.1016/j.echo.2013.10.014 24325962 PMC3922965

[35] Koestenberger M Ravekes W. Right ventricular function parameters in the neonatal population. J Am Soc Echocardiogr. (2012) 25:243–4. doi: 10.1016/j.echo.2011.12.004 22189094

[36] Lang RM Badano LP Mor-Avi V Afilalo J Armstrong A Ernande L. Recommendations for cardiac chamber quantification by echocardiography in adults: an update from the American Society of Echocardiography and the European Association of Cardiovascular Imaging. J Am Soc Echocardiogr. (2015) 28:1–39.e14. doi: 10.1016/j.echo.2014.10.003 25559473

[37] Kurath-Koller S Avian A Cantinotti M Burmas A Grangl G Schweintzger S. Normal pediatric values of the subcostal tricuspid annular plane systolic excursion (S-TAPSE) and its value in pediatric pulmonary hypertension. Can J Cardiol. (2019) 35:899–906. doi: 10.1016/j.cjca.2019.01.019 31292089

[38] Prada G Pustavoitau A Koenig S Mitchell C Stainback RF Diaz-Gomez JL. Focused cardiac ultrasonography for right ventricular size and systolic function. N Engl J Med. (2022) 387:e52. doi: 10.1056/NEJMvcm2004089 36416769

[39] Lai WW Gauvreau K Rivera ES Saleeb S Powell AJ Geva T. Accuracy of guideline recommendations for two-dimensional quantification of the right ventricle by echocardiography. Int J Cardiovasc Imaging. (2008) 24:691–8. doi: 10.1007/s10554-008-9314-4 18438737

[40] Giusca S Dambrauskaite V Scheurwegs C D'Hooge J Claus P Herbots L. Deformation imaging describes right ventricular function better than longitudinal displacement of the tricuspid ring. Heart. (2010) 96:281–8. doi: 10.1136/hrt.2009.171728 19720609

[41] Lee JZ Low SW Pasha AK Howe CL Lee KS Suryanarayana PG. Comparison of tricuspid annular plane systolic excursion with fractional area change for the evaluation of right ventricular systolic function: a meta-analysis. Open Heart. (2018) 5:e000667. doi: 10.1136/openhrt-2017-000667 29387425 PMC5786917

[42] Romanowicz J Ferraro AM Harrington JK Sleeper LA Adar A Levy PT. Pediatric normal values and *Z* score equations for left and right ventricular strain by two-dimensional speckle-tracking echocardiography derived from a large cohort of healthy children. J Am Soc Echocardiogr. (2023) 36:310–23. doi: 10.1016/j.echo.2022.11.006 36414123

[43] Vieillard-Baron A Boissier F Pesenti A. Hemodynamic impact of prone position. Let’s protect the lung and its circulation to improve prognosis. Intensive Care Med. (2023) 49:692–4. doi: 10.1007/s00134-023-07001-2 36820879

[44] Jozwiak M Teboul JL Anguel N Persichini R Silva S Chemla D. Beneficial hemodynamic effects of prone positioning in patients with acute respiratory distress syndrome. Am J Respir Crit Care Med. (2013) 188:1428–33. doi: 10.1164/rccm.201303-0593OC 24102072

[45] Vieillard-Baron A Charron C Caille V Belliard G Page B Jardin F. Prone positioning unloads the right ventricle in severe ARDS. Chest. (2007) 132:1440–6. doi: 10.1378/chest.07-1013 17925425

[46] Coleman RD Chartan CA Mourani PM. Intensive care management of right ventricular failure and pulmonary hypertension crises. Pediatr Pulmonol. (2021) 56:636–48. doi: 10.1002/ppul.24776 33561307

[47] Abman SH Hansmann G Archer SL Ivy DD Adatia I Chung WK. Pediatric pulmonary hypertension: guidelines from the American Heart Association and American Thoracic Society. Circulation. (2015) 132:2037–99. doi: 10.1161/CIR.0000000000000329 26534956

[48] Hansmann G Koestenberger M Alastalo TP Apitz C Austin ED Bonnet D. 2019 updated consensus statement on the diagnosis and treatment of pediatric pulmonary hypertension: The European Pediatric Pulmonary Vascular Disease Network (EPPVDN), endorsed by AEPC, ESPR and ISHLT. J Heart Lung Transplant. (2019) 38:879–901. doi: 10.1016/j.healun.2019.06.022 31495407

[49] Konstam MA Kiernan MS Bernstein D Bozkurt B Jacob M Kapur NK. Evaluation and management of right-sided heart failure: a scientific statement from the American Heart Association. Circulation. (2018) 137:e578–622. doi: 10.1161/CIR.0000000000000560 29650544

[50] Siehr SL Feinstein JA Yang W Peng LF Ogawa MT Ramamoorthy C. Hemodynamic effects of phenylephrine, vasopressin, and epinephrine in children with pulmonary hypertension: a pilot study. Pediatr Crit Care Med. (2016) 17:428–37. doi: 10.1097/PCC.0000000000000716 27144689

[51] Evora PR Pearson PJ Schaff HV. Arginine vasopressin induces endothelium-dependent vasodilatation of the pulmonary artery. V_1_-receptor-mediated production of nitric oxide. Chest. (1993) 103:1241–5. doi: 10.1378/chest.103.4.1241 8131474

[52] Banerjee D Haddad F Zamanian RT Nagendran J. Right ventricular failure: a novel era of targeted therapy. Curr Heart Fail Rep. (2010) 7:202–11. doi: 10.1007/s11897-010-0031-7 20890792

[53] Marenzi G Lauri G Grazi M Assanelli E Campodonico J Agostoni P. Circulatory response to fluid overload removal by extracorporeal ultrafiltration in refractory congestive heart failure. J Am Coll Cardiol. (2001) 38:963–8. doi: 10.1016/s0735-1097(01)01479-6 11583865

[54] Rimensberger PC Cheifetz IM Pediatric Acute Lung Injury Consensus Conference Group. Ventilatory support in children with pediatric acute respiratory distress syndrome: proceedings from the Pediatric Acute Lung Injury Consensus Conference. Pediatr Crit Care Med. (2015) 16:S51–60. doi: 10.1097/PCC.0000000000000433 26035364

[55] Guérin C Reignier J Richard JC Beuret P Gacouin A Boulain T. Prone positioning in severe acute respiratory distress syndrome. N Engl J Med. (2013) 368:2159–68. doi: 10.1056/NEJMoa1214103 23688302

[56] Guérin C Albert RK Beitler J Gattinoni L Jaber S Marini JJ. Prone position in ARDS patients: why, when, how and for whom. Intensive Care Med. (2020) 46:2385–96. doi: 10.1007/s00134-020-06306-w 33169218 PMC7652705

[57] Papazian L Munshi L Guérin C. Prone position in mechanically ventilated patients. Intensive Care Med. (2022) 48:1062–5. doi: 10.1007/s00134-022-06731-z 35652920 PMC9160174

[58] Gattinoni L Busana M Giosa L Macrì MM Quintel M. Prone positioning in acute respiratory distress syndrome. Semin Respir Crit Care Med. (2019) 40:94–100. doi: 10.1055/s-0039-1685180 31060091

[59] Boesing C Graf PT Schmitt F Thiel M Pelosi P Rocco PRM. Effects of different positive end-expiratory pressure titration strategies during prone positioning in patients with acute respiratory distress syndrome: a prospective interventional study. Crit Care. (2022) 26:82. doi: 10.1186/s13054-022-03956-8 35346325 PMC8962042

[60] Park J Lee HY Lee J Lee SM. Effect of prone positioning on oxygenation and static respiratory system compliance in COVID-19 ARDS vs. non-COVID ARDS. Respir Res. (2021) 22:220. doi: 10.1186/s12931-021-01819-4 34362368 PMC8343350

[61] Paternot A Repessé X Vieillard-Baron A. Rationale and description of right ventricle-protective ventilation in ARDS. Respir Care. (2016) 61:1391–6. doi: 10.4187/respcare.04943 27484108

[62] Katira BH Giesinger RE Engelberts D Zabini D Kornecki A Otulakowski G. Adverse heart-lung interactions in ventilator-induced lung injury. Am J Respir Crit Care Med. (2017) 196:1411–21. doi: 10.1164/rccm.201611-2268OC 28795839

[63] Puybasset L Cluzel P Gusman P Grenier P Preteux F Rouby JJ. Regional distribution of gas and tissue in acute respiratory distress syndrome. I. Consequences for lung morphology. CT Scan ARDS Study Group. Intensive Care Med. (2000) 26:857–69. doi: 10.1007/s001340051274 10990099

[64] Palmer RM Ashton DS Moncada S. Vascular endothelial cells synthesize nitric oxide from L-arginine. Nature. (1988) 333:664–6. doi: 10.1038/333664a0 3131684

[65] Gebistorf F Karam O Wetterslev J Afshari A. Inhaled nitric oxide for acute respiratory distress syndrome (ARDS) in children and adults. Cochrane Database Syst Rev. (2016) 2016:CD002787. doi: 10.1002/14651858.CD002787.pub3 27347773 PMC6464789

[66] Domingo LT Ivy DD Abman SH Grenolds AM MacLean JT Breaux JA. Novel use of riociguat in infants with severe pulmonary arterial hypertension unable to wean from inhaled nitric oxide. Front Pediatr. (2022) 10:1014922. doi: 10.3389/fped.2022.1014922 36533232 PMC9751701

[67] Löllgen H Drexler H. Use of inotropes in the critical care setting. Crit Care Med. (1990) 18:S61–15. doi: 10.1097/00003246-199001002-00011 2136722

[68] Faritous SZ Zaree SR Morshedizad Z Jalali AH Mahani SM Gholampour M. The effect of calcium gluconate administration during cardiopulmonary bypass on hemodynamic variables in infants undergoing open-heart surgery. Egypt Heart J. (2022) 74:29. doi: 10.1186/s43044-022-00266-w 35416549 PMC9006523

[69] Elidrissy AT Munawarah M Alharbi KM. Hypocalcemic rachitic cardiomyopathy in infants. J Saudi Heart Assoc. (2013) 25:25–33. doi: 10.1016/j.jsha.2012.11.003 24174842 PMC3809507

[70] Sanyal D Raychaudhuri M. Infants with dilated cardiomyopathy and hypocalcemia. Indian J Endocrinol Metab. (2013) 17:S221–3. doi: 10.4103/2230-8210.119578 24251165 PMC3830311

[71] Silvetti S Belletti A Bianzina S Momeni M. Effect of Levosimendan treatment in pediatric patients with cardiac dysfunction: an update of a systematic review and meta-analysis of randomized controlled trials. J Cardiothorac Vasc Anesth. (2022) 36:657–64. doi: 10.1053/j.jvca.2021.09.018 34656399

[72] Gist KM Mizuno T Goldstein SL Vinks A. Retrospective evaluation of milrinone pharmacokinetics in children with kidney injury. Ther Drug Monit. (2015) 37:792–6. doi: 10.1097/FTD.0000000000000214 25860636

[73] Belenkie I Horne SG Dani R Smith ER Tyberg JV. Effects of aortic constriction during experimental acute right ventricular pressure loading. Further insights into diastolic and systolic ventricular interaction. Circulation. (1995) 92:546–54. doi: 10.1161/01.cir.92.3.546 7634469

[74] Holmes CL Patel BM Russell JA Walley KR. Physiology of vasopressin relevant to management of septic shock. Chest. (2001) 120:989–1002. doi: 10.1378/chest.120.3.989 11555538

[75] Valentine SL Nadkarni VM Curley MA Pediatric Acute Lung Injury Consensus Conference Group. Nonpulmonary treatments for pediatric acute respiratory distress syndrome: proceedings from the Pediatric Acute Lung Injury Consensus Conference. Pediatr Crit Care Med. (2015) 16:S73–85. doi: 10.1097/PCC.0000000000000435 26035367

[76] Haddad F Doyle R Murphy DJ Hunt SA. Right ventricular function in cardiovascular disease, part ii: pathophysiology, clinical importance, and management of right ventricular failure. Circulation. (2008) 117:1717–31. doi: 10.1161/CIRCULATIONAHA.107.653584 18378625

[77] Vonk Noordegraaf A Westerhof BE Westerhof N. The relationship between the right ventricle and its load in pulmonary hypertension. J Am Coll Cardiol. (2017) 69:236–43. doi: 10.1016/j.jacc.2016.10.047 28081831

[78] Dell'Italia LJ Starling MR Blumhardt R Lasher JC O'Rourke RA. Comparative effects of volume loading, dobutamine, and nitroprusside in patients with predominant right ventricular infarction. Circulation. (1985) 72:1327–35. doi: 10.1161/01.cir.72.6.1327 4064277

[79] Ellison DH. Diuretic therapy and resistance in congestive heart failure. Cardiology. (2001) 96:132–43. doi: 10.1159/000047397 11805380

[80] Boerrigter G Burnett JC. Cardiorenal syndrome in decompensated heart failure: prognostic and therapeutic implications. Curr Heart Fail Rep. (2004) 1:113–20. doi: 10.1007/s11897-004-0020-9 16036034

[81] Maratta C Potera RM van Leeuwen G Castillo Moya A Raman L Annich GM. Extracorporeal Life Support Organization (ELSO): 2020 Pediatric Respiratory ELSO Guideline. ASAIO J. (2020) 66:975–9. doi: 10.1097/MAT.0000000000001223 32701626

[82] Brown G Moynihan KM Deatrick KB Hoskote A Sandhu HS Aganga D. Extracorporeal Life Support Organization (ELSO): guidelines for pediatric cardiac failure. ASAIO J. (2021) 67:463–75. doi: 10.1097/MAT.0000000000001431 33788796

[83] Price LC McAuley DF Marino PS Finney SJ Griffiths MJ Wort SJ. Pathophysiology of pulmonary hypertension in acute lung injury. Am J Physiol Lung Cell Mol Physiol. (2012) 302:L803–15. doi: 10.1152/ajplung.00355.2011 22246001 PMC3362157

[84] Grant C Richards JB Frakes M Cohen J Wilcox SR. ECMO and right ventricular failure: review of the literature. J Intensive Care Med. (2021) 36:352–60. doi: 10.1177/0885066619900503 31964208

[85] Lazzeri C Bonizzoli M Cianchi G Batacchi S Guetti C Cozzolino M. Right ventricular dysfunction and pre implantation vasopressors in refractory ARDS supported by VV-ECMO. Heart Lung Circ. (2018) 27:1483–8. doi: 10.1016/j.hlc.2017.10.011 29128166

[86] Bunge JJH Caliskan K Gommers D Reis MD. Right ventricular dysfunction during acute respiratory distress syndrome and veno-venous extracorporeal membrane oxygenation. J Thorac Dis. (2018) 10:S674–82. doi: 10.21037/jtd.2017.10.75 29732186 PMC5911554

[87] Srivastava MC Ramani GV Garcia JP Griffith BP Uber PA Park MH. Veno-venous extracorporeal membrane oxygenation bridging to pharmacotherapy in pulmonary arterial hypertensive crisis. J Heart Lung Transplant. (2010) 29:811–3. doi: 10.1016/j.healun.2010.02.005 20417127

[88] Schmidt M Tachon G Devilliers C Muller G Hekimian G Bréchot N. Blood oxygenation and decarboxylation determinants during venovenous ECMO for respiratory failure in adults. Intensive Care Med. (2013) 39:838–46. doi: 10.1007/s00134-012-2785-8 23291732

[89] Morimont P Guiot J Desaive T Tchana-Sato V Janssen N Cagnina A. Veno-venous extracorporeal Co_2_ removal improves pulmonary hemodynamics in a porcine ARDS model. Acta Anaesthesiol Scand. (2015) 59:448–56. doi: 10.1111/aas.12497 25736472

[90] Jenks CL Raman L Dalton HJ. Pediatric extracorporeal membrane oxygenation. Crit Care Clin. (2017) 33:825–41. doi: 10.1016/j.ccc.2017.06.005 28887930

[91] Cevasco M Takayama H Ando M Garan AR Naka Y Takeda K. Left ventricular distension and venting strategies for patients on venoarterial extracorporeal membrane oxygenation. J Thorac Dis. (2019) 11:1676–83. doi: 10.21037/jtd.2019.03.29 31179113 PMC6531683

[92] Brodie D Bacchetta M. Extracorporeal membrane oxygenation for ARDS in adults. N Engl J Med. (2011) 365:1905–14. doi: 10.1056/NEJMct1103720 22087681

[93] Schmidt M Stewart C Bailey M Nieszkowska A Kelly J Murphy L. Mechanical ventilation management during extracorporeal membrane oxygenation for acute respiratory distress syndrome: a retrospective international multicenter study. Crit Care Med. (2015) 43:654–64. doi: 10.1097/CCM.0000000000000753 25565460

[94] Chad T Yusuff H Zochios V Pettenuzzo T Fan E Schmidt M. Right ventricular injury increases mortality in patients with acute respiratory distress syndrome on veno-venous extracorporeal membrane oxygenation: a systematic review and meta-analysis. ASAIO J. (2023) 69:e14–22. doi: 10.1097/MAT.0000000000001854 36375040

[95] Douflé G Roscoe A Billia F Fan E. Echocardiography for adult patients supported with extracorporeal membrane oxygenation. Crit Care. (2015) 19:326. doi: 10.1186/s13054-015-1042-2 26428448 PMC4591622

[96] Krishnan S Schmidt GA. Hemodynamic monitoring in the extracorporeal membrane oxygenation patient. Curr Opin Crit Care. (2019) 25:285–91. doi: 10.1097/MCC.0000000000000602 30865613

[97] Lazzeri C Cianchi G Bonizzoli M Batacchi S Terenzi P Bernardo P. Right ventricle dilation as a prognostic factor in refractory acute respiratory distress syndrome requiring veno-venous extracorporeal membrane oxygenation. Minerva Anestesiol. (2016) 82:1043–9. 26957118

[98] Ortiz F Brunsvold ME Bartos JA. Right ventricular dysfunction and mortality after cannulation for venovenous extracorporeal membrane oxygenation. Crit Care Explor. (2020) 2:e0268. doi: 10.1097/CCE.0000000000000268 33196050 PMC7655090

[99] Guervilly C Hraiech S Gariboldi V Xeridat F Dizier S Toesca R. Prone positioning during veno-venous extracorporeal membrane oxygenation for severe acute respiratory distress syndrome in adults. Minerva Anestesiol. (2014) 80:307–13. 24257150

[100] Rilinger J Zotzmann V Bemtgen X Schumacher C Biever PM Duerschmied D. Prone positioning in severe ARDS requiring extracorporeal membrane oxygenation. Crit Care. (2020) 24:397. doi: 10.1186/s13054-020-03110-2 32641155 PMC7341706

[101] Mebazaa A Karpati P Renaud E Algotsson L. Acute right ventricular failure-from pathophysiology to new treatments. Intensive Care Med. (2004) 30:185–96. doi: 10.1007/s00134-003-2025-3 14618229

[102] Schmidt M Bailey M Kelly J Hodgson C Cooper DJ Scheinkestel C. Impact of fluid balance on outcome of adult patients treated with extracorporeal membrane oxygenation. Intensive Care Med. (2014) 40:1256–66. doi: 10.1007/s00134-014-3360-2 24934814 PMC7094895

[103] El Banayosy AM El Banayosy A Brewer JM Mihu MR Chidester JM Swant LV. The ProtekDuo for percutaneous V-P and V-VP ECMO in patients with COVID-19 ARDS. Int J Artif Organs. (2022) 45:1006–12. doi: 10.1177/03913988221121355 36085584 PMC9465053

[104] Bunge JJH Diaby S Valle AL Bakker J Gommers D Vincent JL. Safety and efficacy of beta-blockers to improve oxygenation in patients on veno-venous ECMO. J Crit Care. (2019) 53:248–52. doi: 10.1016/j.jcrc.2019.06.024 31295671

[105] Guarracino F Zangrillo A Ruggeri L Pieri M Calabro MG Landoni G. Beta-blockers to optimize peripheral oxygenation during extracorporeal membrane oxygenation: a case series. J Cardiothorac Vasc Anesth. (2012) 26:58–63. doi: 10.1053/j.jvca.2011.05.013 21764329

[106] Beltramo F Khemani RG. Definition and global epidemiology of pediatric acute respiratory distress syndrome. Ann Transl Med. (2019) 7:502. doi: 10.21037/atm.2019.09.31 31728355 PMC6828787

